# Supplementary figures and images for: Genotypic richness predicts phenotypic variation in an endangered clonal plant
Source: PeerJ. 2016 Feb 18;4:e1633. doi: 10.7717/peerj.1633 (PMC4768672; doi:10.7717/peerj.1633)

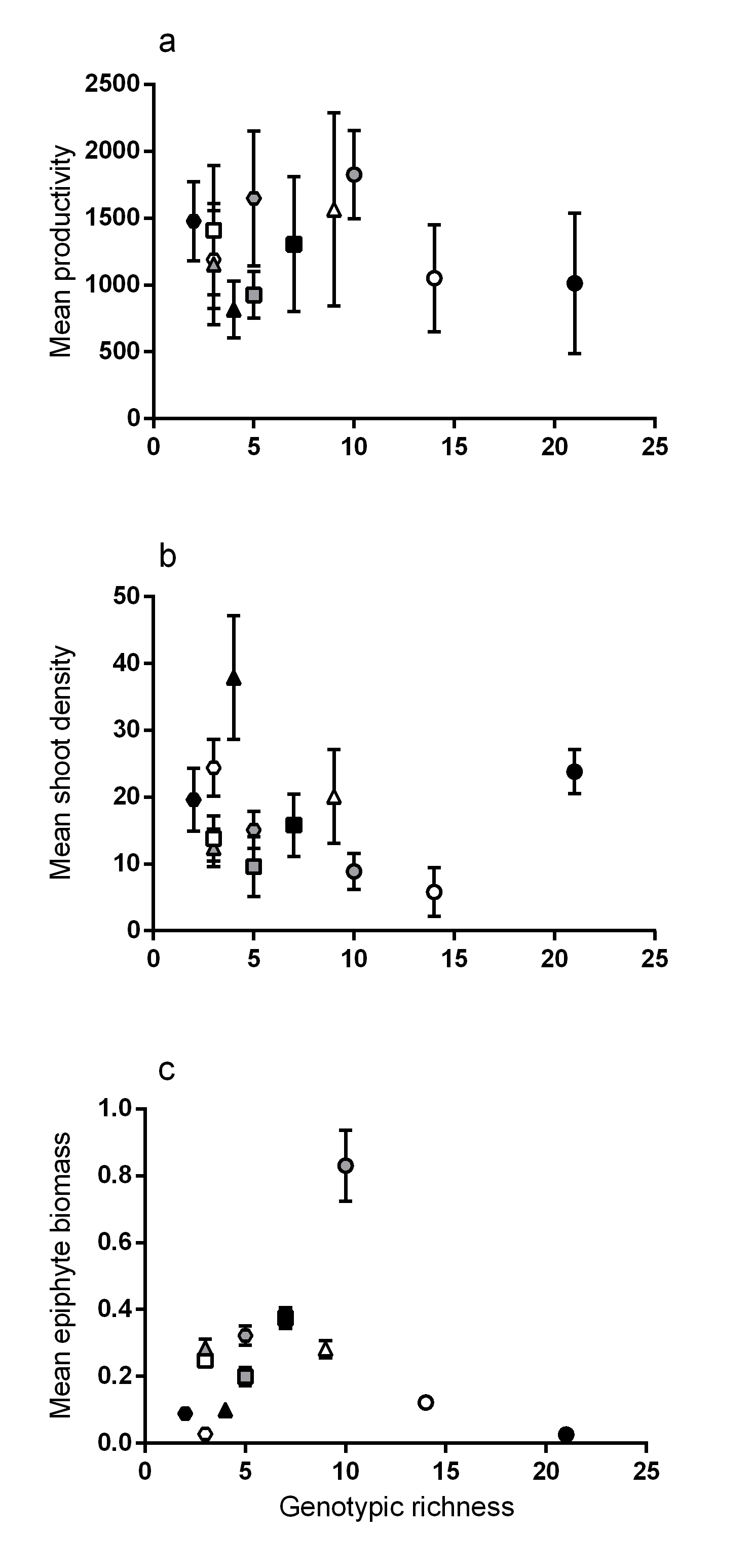

Supplement: Figure S1 — Relationships between genotypic richness and (A) mean productivity (mg dw shoot−1 year−1); (B) mean shoot density (shoots 0.25 m2−1); and (C) mean epiphyte biomass (mg dw shoot−1). There were no significant relationships between genotypic richness and the mean values of any of the above traits (a: R2 = 0.02, P = 0.69; b: R2 = 0.15, P = 0.22; or c: R2 = 0.001, P = 0.96). Different symbols correspond to individual meadows sampled (see Fig. 1). [file peerj-04-1633-s001.png]

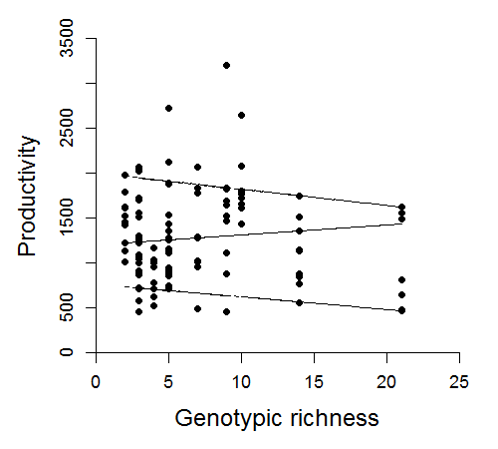

Supplement: Figure S2 — Quantile regression on raw productivity data (mg dw shoot−1 year−1) and genotypic richness for the 90th, 50th and 10th percentile (top, middle and bottom lines). This test is used to look at the dispersion of data points within each grouping, in this case to see whether the capacity for high productivity was greater in more genotypically rich meadows. Here we can see that this is not the case, as there is no significant relationship between productivity and richness in the 90th percentile (t = − 0.44, P = 0.66). Similarly there is no significant relationship in the 50th percentile (t = 0.64, P = 0.52). However, there is a significant negative relationship between genotypic richness and productivity in the 10th percentile (t = − 3.07, P = 0.003). [file peerj-04-1633-s002.png]

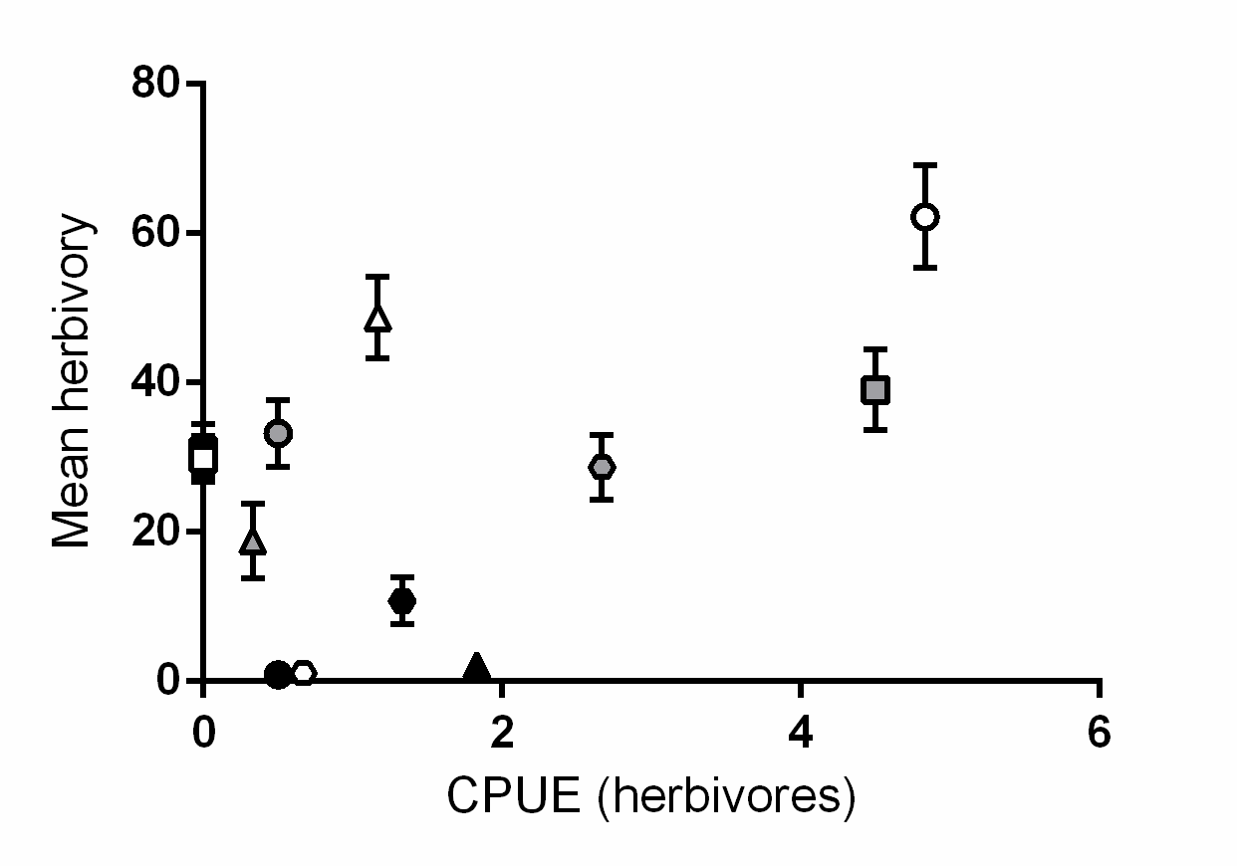

Supplement: Figure S3 — The relationship between the number of herbivorous fish per site (standardised using catch per unit effort) and mean herbivory (mm2 shoot−1). There was no relationship between mean herbivory and fish abundance (R2 = 0.27, P = 0.08). Different symbols correspond to individual meadows sampled (see Fig. 1). [file peerj-04-1633-s003.png]

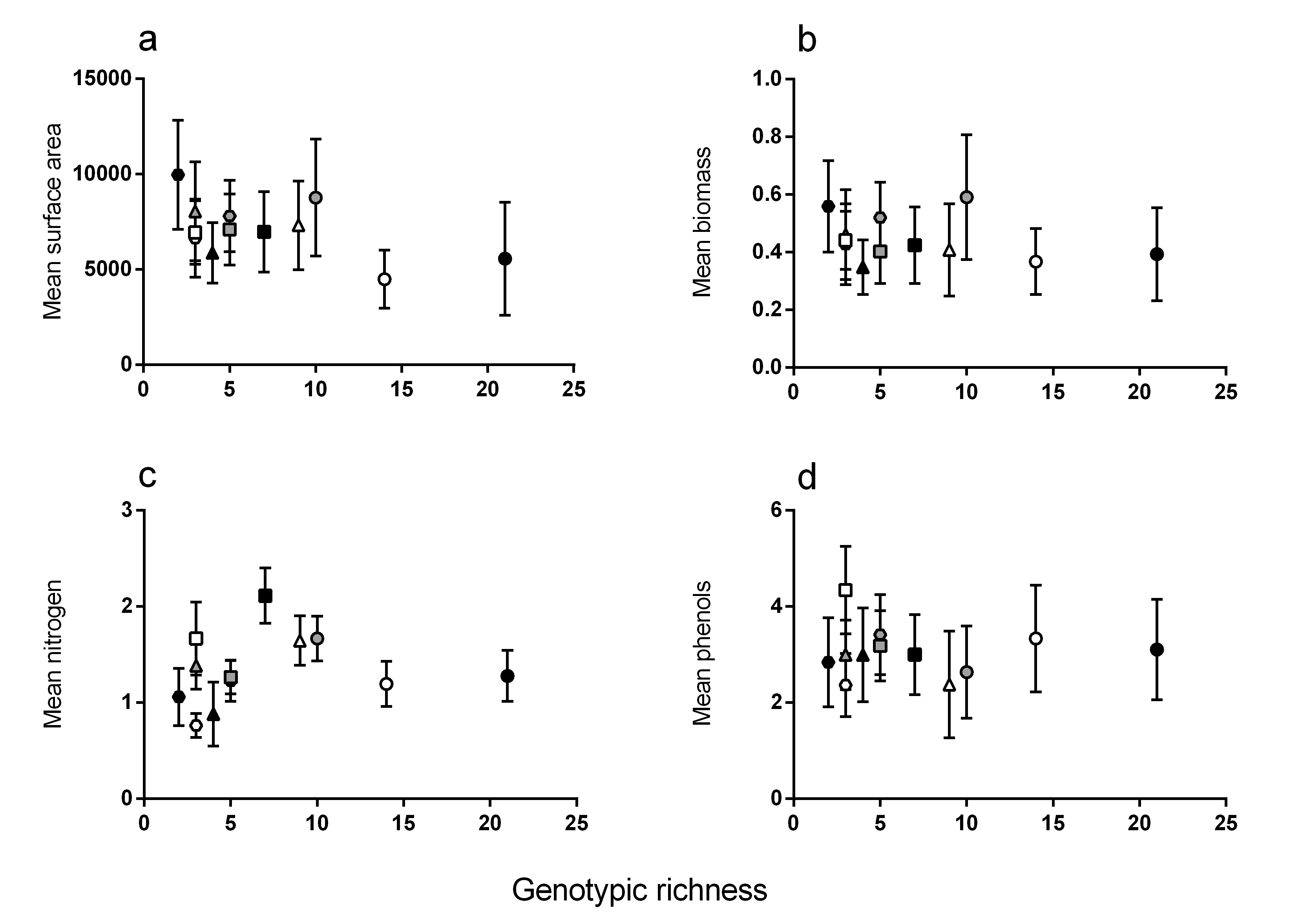

Supplement: Figure S4 — Relationships between genotypic richness and leaf traits: (A) surface area (mm2 shoot−1); (B) biomass (mg dw shoot−1); (C) mean nitrogen (GAE % dw shoot−1); and (D) mean phenols (GAE % dw shoot−1). There were no significant relationships between genotypic richness and the mean values of any of the above traits (a: R2 = 0.28, P = 0.08; b: R2 = 0.07, P = 0.41; c: R2 = 0.02, P = 0.63; or d: R2 = 0.003, P = 0.87). Different symbols correspond to individual meadows sampled (see Fig. 1). [file peerj-04-1633-s004.png]

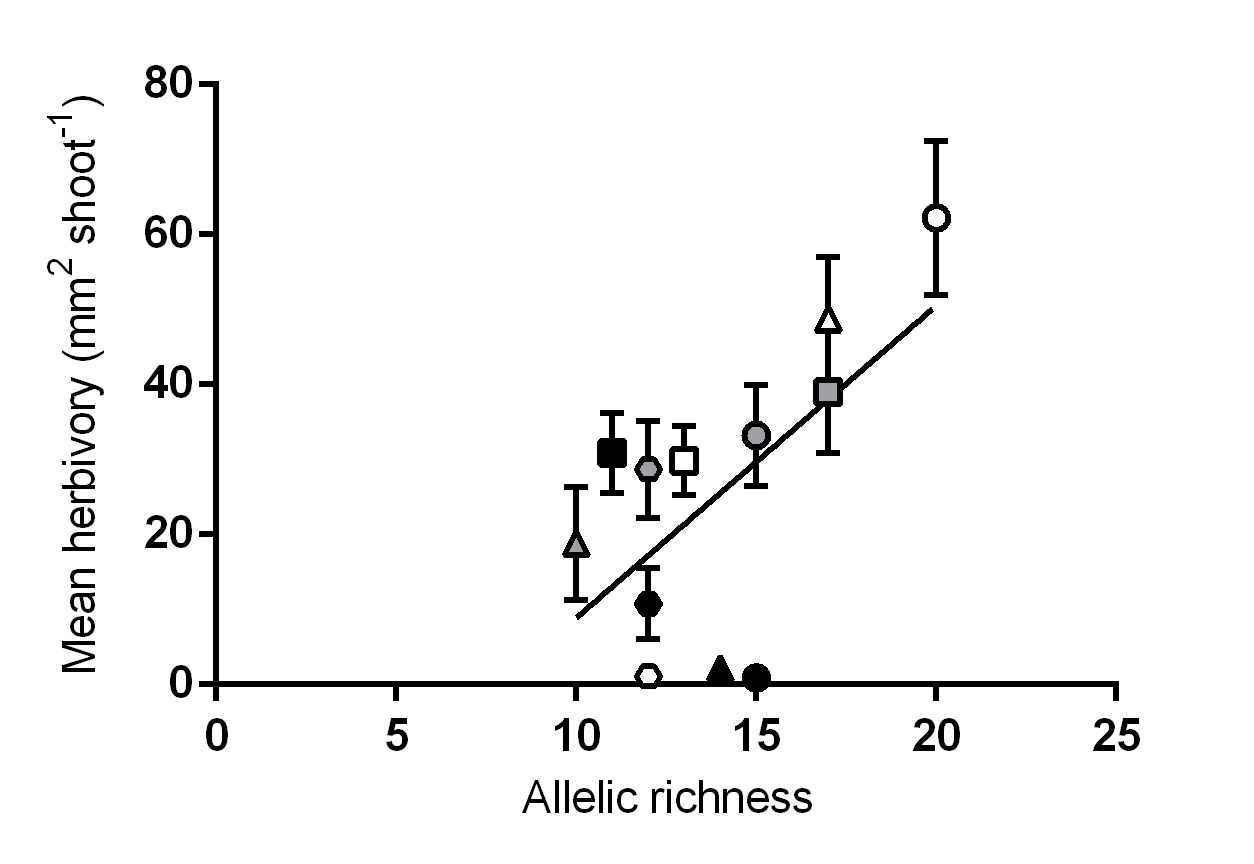

Supplement: Figure S5 — Different symbols correspond to individual meadows sampled (see Fig. 1). [file peerj-04-1633-s005.png]
